# Effects of ^ch^aducanumab‐mediated plaque removal on amyloid‐tau synergy

**DOI:** 10.1002/alz.091508

**Published:** 2025-01-03

**Authors:** Lindsay A Welikovitch, Anastasie Mate de Gerando, Anita Khasnavis, Harshil Bhavsar, Jonah C Meltzer, Luc Buee, Thierry Bussière, Bradley T. T Hyman

**Affiliations:** ^1^ Massachusetts General Hospital, Boston, MA USA; ^2^ Harvard Medical School, Boston, MA USA; ^3^ Université de Lille, Lille, Hauts‐de‐France France; ^4^ Biogen, Cambridge, MA USA

## Abstract

**Background:**

Amyloid‐targeting antibodies have been shown to be remarkably effective at clearing amyloid plaques from the Alzheimer’s disease (AD) brain. To date, preclinical assessments have used animal models that develop only amyloid pathology, whereas AD patients present with tau pathology, neuroinflammation, and other concurrent neuropathologies. Deciphering how successful anti‐amyloid therapies impact the synergistic interplay of amyloid and tau will be critical in determining which secondary disease processes can be slowed, interrupted, or reversed by amyloid‐targeting immunotherapies.

**Method:**

To establish whether interrupting Aβ plaque deposition or clearing existing plaques could halt the progression of tau pathology and downstream disease events, we treated APP/PS1xTau22 mice with a murine chimeric analog of Aducanumab (^ch^Adu) targeting human oligomeric and fibrillar Aβ species. Equal concentrations of ^ch^Adu and isotype Control IgG were administered weekly for 12 weeks by intraperitoneal injection at a dose of 30mg/kg beginning at 6 months of age.

**Result:**

Following 3 months of immunotherapy, APP/PS1xTau22 mice treated with ^ch^Adu had lower Aβ plaque burden in both the hippocampus and cortex. Although amyloid expression accelerates the formation of tau neurofibrillary tangles as early as 6 months of age in the APP/PS1xTau22 transgenic model, inhibiting further plaque deposition using ^ch^Adu had no effect on the abundance of AT8^+^ or AT100^+^ neurofibrillary lesions in any brain region analyzed. While regional phospho‐tau deposition was similar between isotype‐IgG and ^ch^Adu‐treated animals, we observed a reduction in the number of AT8^+^and SMI312^+^ axonal dystrophies in mice that had undergone 3 months of immunotherapy.

**Conclusion:**

Within a more‐complex neuropathological environment, amyloid immunotherapy influences plaque‐related secondary disease events, but does not directly impact tau deposition in the APP/PS1xTau22 mouse model. Identifying which aspects of the AD pathology remain resistant to amyloid immunotherapy will provide insight into how mono‐ or combination therapies can be developed or improved.

